# Statins research unfinished saga: desirability versus feasibility

**DOI:** 10.1186/1475-2840-4-8

**Published:** 2005-06-07

**Authors:** Enrique Z Fisman, Yehuda Adler, Alexander Tenenbaum

**Affiliations:** 1 The Sackler Faculty of Medicine, Tel-Aviv University, 69978 Ramat-Aviv, Tel-Aviv, Israel; 2 Cardiac Rehabilitation Institute, Sheba Medical Center, 52621 Tel-Hashomer, Israel

**Keywords:** coronary artery disease, diabetes, hyperlipidemia, statins, trials

## Abstract

Drugs in the same class are generally thought to be therapeutically equivalent because of similar mechanisms of action (the so-called "class effect"). However, statins differ in multiple characteristics, including liver and renal metabolism, half-life, effects on several serum lipid components, bioavailability and potency. Some are fungal derivatives, and others are synthetic compounds. The percentage absorption of an oral dose, amount of protein binding, degree of renal excretion, hydrophilicity, and potency on a weight basis is variable. These differences may be even greater in diabetic patients, who may present diabetes-induced abnormalities in P450 isoforms and altered hepatic metabolic pathways. Thus, it is obvious that head-to-head comparisons between different statins are preferable than trial-to-trial comparisons. Such assessments are of utmost importance, especially in cases in which specific populations with a distinct lipid profile and altered metabolic pathways, like diabetics, are studied. It should be specially pinpointed that patients with metabolic syndrome and diabetes constitute also a special population regarding their atherogenic dyslipidemia, which is usually associated with low HDL-cholesterol, hypertriglyceridemia and predominance of small dense LDL-cholesterol. Therefore, these patients may benefit from fibrates or combined statin/fibrate treatment. This policy is not accomplished since in the real world things are more complex. Trials would require very large sample sizes and long-term follow-up to detect significant differences in myocardial infarction or death between two different statins. Moreover, the fact that new compounds are under several phases of research and development represents an additional drawback for performing the trials. Ideally, head-to-head trials regarding clinically important outcomes should be conducted for all drugs. Nonetheless, the desirability of performing such trials, which epitomize modern evidence-based medicine, is frequently superseded by the feasibility dictated by pragmatic and economic circumstances. In the latter case, in absence of solid systematic documentation of comparable health benefits and long-term safety, both researchers and practicing physicians should allude to the weight of scientific endorsement behind the arguments and seek for the possible strengths and weaknesses intrinsic to each specific study. In any case, conclusions based on surrogate endpoints cannot completely substitute head-to-head comparisons regarding patients' outcome.

## 

During the last fifteen years large-scale clinical trials were conducted aiming to determine the antihyperlipidemic activity of several 3-hydroxy-3-methylglutaryl coenzyme A reductase inhibitors (statins). These studies included the Scandinavian Simvastatin Survival Study (4S) [1], the West of Scotland Coronary Prevention Study (WOSCOPS) [2], the Expanded Clinical Evaluation of Lovastatin (EXCEL) study [3], among others. Several important concepts emerged from these studies. It was shown that lipid-lowering pharmacotherapy is justified for patients with hypercholesterolemia who are at risk of coronary artery disease (CAD). Lipid-lowering medication produced significant reductions in death from CAD and non-fatal myocardial infarction (MI), all cardiovascular death and all-cause mortality, clearly supporting the use of statins in such patients. In addition, statins induced modest increases in high-density lipoprotein cholesterol, and decreases in triglycerides [1-3]. No direct comparisons between these drugs were available at this stage. Thus, questions regarding the relative superiority and/or safety of a given compound remained unanswered. These queries have need of an appropriate response.

## Head-to-head comparisons

Following the pioneering initial studies, more recent head-to-head comparative parallel-group trials unveiled additional features of this type of drugs. In 2001, the Reversal of Atherosclerosis with Aggressive Lipid Lowering (REVERSAL) trial demonstrated that atorvastatin 80 mg/day partially reversed carotid intima-media thickness in patients with familial hypercholesterolemia, as compared with simvastatin 40 mg/day [4].

Furthermore, the Atorvastatin versus Simvastatin on Atherosclerosis Progression (ASAP) trial study showed that high-dose atorvastatin delayed progression of coronary atherosclerosis, as assessed by intravascular ultrasound, while 40 mg/day pravastatin did not [5]. These trials demonstrated that aggressive statin therapy is more effective in terms of atherosclerosis progression. Importantly, statins have also demonstrated to reduce endothelial dysfunction, inflammation, noxious cytokines concentration and blood thrombogenicity, which all seem to be co-responsible for plaque thrombosis [6,7].

In the Pravastatin or Atorvastatin Evaluation and Infection Therapy (PROVE-IT) trial [8] 4162 patients who had suffered an acute coronary syndrome in the preceding 10 days were randomized to receive pravastatin 40 mg/day *vs *atorvastatin 80 mg/day in addition to gatifloxacin *vs *placebo. The data of the lipid-lowering therapy showed mean low-density lipoprotein (LDL) levels of 95 mg/dl in the pravastatin group, in compliance with present guidelines of clinical practice, and 62 mg/dl in the atorvastatin group. After a follow-up of 2 years, the intensive lipid-lowering group showed a significant reduction in the incidence of the combined end-point of death, MI, unstable angina requiring hospitalization, revascularization and stroke. The PROVE-IT findings were strengthened by the Aggressive Lipid-Lowering Initiation Abates New Cardiac Events (ALLIANCE) study, which confirmed the hypothesis of further benefits with aggressive cholesterol-lowering [9]. In this trial, 2442 patients with coronary artery disease were randomized to achieve LDL levels <80 mg/dl, receiving atorvastatin titrated at doses ≤ 80 mg/day, or standard therapy with the statins and doses chosen by the treating physicians. Intensive lipid-lowering therapy demonstrated a significant reduction in the primary end-point of combined incidence of cardiac death, myocardial infarction, coronary revascularization and unstable angina requiring hospitalization.

However, it should be pinpointed that lipid reduction, decrease of markers of hemostasis and inflammation or reduction in number of atherosclerotic plaques should be viewed as surrogate end points of statins activity. The extent to which these results can be extrapolated to clinically relevant outcomes remains to be established [10]. It is yet unclear whether all statins are equally effective in preventing recurrent MI and death at a long-term follow-up. Drugs in the same class are generally thought to be therapeutically equivalent because of similar mechanisms of action (the so-called "class effect" [11]). In this context, however, statins differ in multiple characteristics, including liver and renal metabolism, half-life, effects on several serum lipid components, bioavailability and potency. In addition, some are fungal derivatives, and others are synthetic compounds. The percentage absorption of an oral dose, amount of protein binding, degree of renal excretion, hydrophilicity, and potency on a weight basis varies among the individual agents [12,13]. Despite these differences, a just published Canadian retrospective cohort study comparing atorvastatin, pravastatin, simvastatin, lovastatin and fluvastatin in patients aged >65 years after their first MI claims that all these statins are equally effective [10]. The study included above 18500 patients who began statin treatment within 90 days after discharge, and the primary end point was the combined outcome of recurrent MI or death from any cause.

## Trial-versus-trial comparisons

These comparisons present the hazard of being potentially misleading because of fundamental "apples-to-oranges" comparisons secondary to differences between trials in patient populations, inclusion criteria, management algorithms, and end-point definitions [14]. Although statins share common main actions, they may have clinically important differences in terms of efficacy and safety. In this context, it should be remembered that as a result of 31 reported cases of fatal rhabdomyolysis, cerivastatin was withdrawn from the market, underscoring the risk of therapeutic interchangeability [15]. Modern pharmacological decision-making and guidelines development often rely on meta-analyses in order to handle the vast amount of clinical information available from clinical trials in cardiovascular medicine. Despite the establishment of standards for reporting meta-analyses, there are also limitations that need to be acknowledged. Statistical testing for heterogeneity of treatment effects across drugs in a class is not a particularly sensitive analysis. Thus, without access to original source data, reliably identifying adverse events can be very difficult [14].

An event illustrating this problem comes from New Zealand. After a government decision to replace simvastatin with fluvastatin as the approved statin for reimbursement, Thomas and Mann [16] investigated the effect of the switch in 126 patients; the lipid concentrations went up in 115 (94%) of the patients after the switch to fluvastatin. In addition, during the 6 months before the switch, there were nine arterial thrombotic events compared with 27 during the same time after the switch (p < 0.05). This study spells out the danger of allowing budgetary arguments about class effects to influence drug selection.

## Statins in diabetic patients

Part of the patients included in studies evaluating statins are diabetics. Both the PROVE-IT [8] and the ALLIANCE [9] trials were performed in populations comprising both diabetic and nondiabetic patients, albeit the percentage of diabetic patients was relatively small, averaging about 20% of the examinees.

Various trials demonstrated improved prognosis and reduction of cardiovascular events in patients with diabetes or impaired fasting glucose taking several statins [17-20]. However, none of these was a head-to-head comparison of two statins regarding their effectiveness in a diabetic population. The recently published study by Berne and Siewert-Delle [21] addresses precisely this point. Rosuvastatin was compared with atorvastatin in type 2 diabetes mellitus for the reduction of LDL-cholesterol. They found that at 4 weeks, 65% of rosuvastatin patients had reached their 2003 European LDL- cholesterol goal (<2.5 mmol/L), compared with 33% of atorvastatin patients (p < 0.0001). Both treatments were similarly well tolerated with no unexpected safety concerns.

The mechanism behind these differences has not yet been elucidated. A possible explanation is the dissimilarity in their hepatic metabolization. Atorvastatin is a relatively lipophilic compound; lipophilic statins are more susceptible to metabolism by the cytochrome P450 system. On the contrary, rosuvastatin is relatively hydrophilic and not significantly metabolized by cytochrome P450 enzymes [22]. Diabetes-induced abnormalities in P450 isoforms were described in both experimental and clinical studies [22-24]. This could explain the better compliance of diabetic patients to the latter drug.

## Lipid-modifying strategy in metabolic syndrome and diabetes: beyond the LDL-cholesterol

Whereas statins remain the useful drug for patients who need to achieve the LDL-cholesterol goal, other lipid-lowering compounds – fibrates – may represent the alternative intervention for subjects with atherogenic dyslipidemia typical for metabolic syndrome and an LDL-cholesterol already close to goal values. An atherogenic dyslipidemia is characterized by elevated levels of triglycerides, reduced levels of high-density lipoprotein cholesterol and a preponderance of small dense LDL-cholesterol particles.

The Adult Treatment Panel III guidelines suggest that because elevated trigycerides are an independent CAD risk factor, some trigyceride-rich lipoproteins, commonly called remnant lipoproteins, must be atherogenic. A novel method for the isolation and quantification of plasma remnants was developed by Nakajima and Nakamura [25]. Specific immunoaffinity-based gel is incubated with plasma, which results in binding of high-density lipoproteins (HDL), LDL, and the majority of very low-density lipoproteins (VLDL) particles to the gel. *Unbound lipoproteins *are quantified on the basis of their cholesterol content; this is termed the remnant-like particles cholesterol (RLP-C) concentration. It is established that elevated plasma RLP-C levels are associated with endothelial dysfunction, a marker for atherosclerotic disease. Patients with established coronary heart disease present elevated plasma levels of RLP-C. Elevated levels of plasma RLP-C were predictive of future coronary events in patients with CAD independently of other risk factors [26].

Extensive evidence from intervention trials has been presented to demonstrate that "old" statin treatment results in reduced cardiovascular morbidity and mortality. By contrast, statin treatment has not been associated consistently with reduction in plasma RLP-C levels. In general, however, it can be concluded that RLP-C lowering by statins depends on their ability to reduce triglyceride levels. Therefore, among all currently clinically available statins only atorvastatin and rosuvastatin have potential to reduce RLP-C levels [27].

In addition, the concomitant use of fibrate and statin seems to be attractive in patients whose LDL-cholesterol is controlled by statin but whose HDL-cholesterol and/or triglyceride levels are still inappropriate [28]. A combination statin/fibrate may be necessary to control all lipid abnormalities in patients with metabolic syndrome and diabetes. Safety concerns about some fibrates such as gemfibrozil may lead to exaggerate precautions regarding fibrate administration and therefore diminish the use of these agents. However, other fibrates- such as bezafibrate and fenofibrate – appear to be safer and better tolerated [29-32].

The combination of fibrate with statin which are not mainly metabolized within the liver by the cytochrome P450 system (like pravastatin or fluvastatin) may be even less hazardous. We believe that a proper coadministration of statins and fibrates in some cases, selected on basis of their safety, could be more effective in achieving a comprehensive lipid control as compared with monotherapy. An alternative sort of future combination may hypothetically be represented by ezetimibe and fibrate. Ezetimibe represents the newest class of lipid-modifying agents. It exerts its cholesterol-lowering effect by inhibiting the absorption of both the dietary cholesterol and biliary cholesterol at the brush border of the small intestine. Because of its distinct mechanism of action, ezetimibe appears to be most useful as part of combination therapy with other lipid-modifying agents rather than as monotherapy. Ezetimibe is approved currently for use alone or with statins, and initial study with fibrates are promising [32-34].

## Desirability and feasibility

Prospective randomized parallel-group head-to-head comparisons between different drugs were not performed during the early stages of statins human use. The main reason was the unwillingness of industry sponsors to take on the risk of failing to show their drug is noninferior to another agent in the same class [14]. Moreover, such a study may potentially became a menacing 'boomerang" if it demonstrates that the competitor's drug is preferable, as it actually happened in the PROVE-IT trial [8]. While statistical techniques for estimating the comparative therapeutic efficacy of "competing" compounds using indirect methods have been proposed, the soundness of these adjusted indirect comparisons is limited, and depends on the accuracy and similarity of the included trials [35].

Thus, it seems even needless to state that head-to-head comparisons are preferable. It would be preferable that every drug (and indeed every dose and every formulation [36]) be evaluated in randomized comparative trials with active comparators from the same class for their effects on clinically important outcomes. This is not accomplished since. Regretfully, in the real world things are more complex.

Pharmaceutical companies spend millions of dollars to convince consumers and doctors that their products are superior to competitor brands; however, these companies cannot be compelled to design and finance trials in which a future monetary profit is dubious.

In addition, trials would require very large sample sizes and long-term follow-up to detect significant differences in myocardial infarction or death between two different statins [35,36]. Moreover, the fact that new compounds, like the presumably potent pitavastatin [37], are under several phases of research and development represents an additional drawback for performing the trials.

The assertion that there really does not appear to be much difference across statins in terms of their effectiveness and they appear to be similar one to another (in a study which did not include rosuvastatin) [10] is seriously defied by head-to-head comparisons performed so far [5-9,21,38]. Such assessments are of utmost importance, especially in cases in which specific populations with a distinct lipid profile and altered metabolic pathways [22-24], like diabetics, are studied.

Ideally, head-to-head trials regarding clinically importantoutcomes should be conducted for all drugs. Nonetheless, the desirability of performing such trials, which epitomize modern evidence-based medicine, is frequently superseded by the feasibility dictated by pragmatic and economic circumstances. In absence of solid systematic documentation of comparable health benefits and long-term safety, both researchers and practicing physicians should allude to the weight of scientific endorsement behind the arguments and seek for the possible strengths and weaknesses intrinsic to each specific study. In addition, surrogate end points, like LDL-cholesterol level, allow conducting shorter and smaller trials with acceptable level of credibility regarding clinical significance of the new compound. A surrogate outcome will be reliable only if there is a validated causal connection between change in surrogate and change in the clinically important outcomes (like morbidity and mortality), and if the surrogate reflects most of the effects of treatment on a specific outcome. The recent paper of Berne and Siewert-Delle [21] denotes a good example for an accurate approach using a relatively reliable (LDL-cholesterol level) surrogate end-point. However, conclusions based on surrogate endpoints can not completely substitute head-to-head trials, particularly in relation to the magnitude of potential benefits in clinical practice.

## List of abbreviations used

ALLIANCE – Aggressive Lipid-Lowering Initiation Abates New Cardiac Events

ASAP – Atorvastatin versus Simvastatin on Atherosclerosis Progression

CAD – coronary artery disease

EXCEL – Expanded Clinical Evaluation of Lovastatin

HDL – high-density lipoproteins

LDL low-density lipoprotein

MI – myocardial infarction

PROVE-IT – Pravastatin or Atorvastatin Evaluation and Infection Therapy

REVERSAL – Reversal of Atherosclerosis with Aggressive Lipid Lowering

RLP-C – remnant-like particles cholesterol

VLDL – very low-density lipoproteins

WOSCOPS – West of Scotland Coronary Prevention Study

4S – Scandinavian Simvastatin Survival Study

## Competing interests

The author(s) declare that they have no competing interests.

## Authors' contributions

All authors contributed equally in the conception and drafting of the manuscript.
